# Valorization of polyphenolic compounds from food industry by-products for application in polysaccharide-based nanoparticles

**DOI:** 10.3389/fnut.2023.1144677

**Published:** 2023-05-24

**Authors:** Thiécla Katiane Osvaldt Rosales, João Paulo Fabi

**Affiliations:** ^1^Department of Food Science and Experimental Nutrition, School of Pharmaceutical Science, University of São Paulo, São Paulo, SP, Brazil; ^2^Food Research Center (FoRC), CEPID-FAPESP (Research, Innovation and Dissemination Centers, São Paulo Research Foundation), São Paulo, SP, Brazil; ^3^Food and Nutrition Research Center (NAPAN), University of São Paulo, São Paulo, SP, Brazil

**Keywords:** antioxidants, bioactive compounds, dietary polyphenols, food industry, dietary supplements, polysaccharides, nanoencapsulation, sustainability

## Abstract

In the last decades, evidence has indicated the beneficial properties of dietary polyphenols. *In vitro* and *in vivo* studies support that the regular intake of these compounds may be a strategy to reduce the risks of some chronic non-communicable diseases. Despite their beneficial properties, they are poorly bioavailable compounds. Thus, the main objective of this review is to explore how nanotechnology improves human health while reducing environmental impacts with the sustainable use of vegetable residues, from extraction to the development of functional foods and supplements. This extensive literature review discusses different studies based on the application of nanotechnology to stabilize polyphenolic compounds and maintain their physical–chemical stability. Food industries commonly generate a significant amount of solid waste. Exploring the bioactive compounds of solid waste has been considered a sustainable strategy in line with emerging global sustainability needs. Nanotechnology can be an efficient tool to overcome the challenge of molecular instability, especially using polysaccharides such as pectin as assembling material. Complex polysaccharides are biomaterials that can be extracted from citrus and apple peels (from the juice industries) and constitute promising wall material stabilizing chemically sensitive compounds. Pectin is an excellent biomaterial to form nanostructures, as it has low toxicity, is biocompatible, and is resistant to human enzymes. The potential extraction of polyphenols and polysaccharides from residues and their inclusion in food supplements may be a possible application to reduce environmental impacts and constitutes an approach for effectively including bioactive compounds in the human diet. Extracting polyphenolics from industrial waste and using nanotechnology may be feasible to add value to food by-products, reduce impacts on nature and preserve the properties of these compounds.

## 1. Introduction

Polyphenolic compounds in foods, supplements, and pharmaceuticals have been gaining interest due to their health benefits, reducing the risks of developing chronic non-communicable diseases (1–3). These natural compounds from the secondary metabolism of plants occur in a wide range of plant species, and regular consumption is highly encouraged, mainly due to their antioxidant properties (4–6). Extracting polyphenolic compounds from residues of food industries and by-products is a viable option to minimize environmental impacts (7, 8). In addition, including natural antioxidants in functional foods and developing dietetic supplements constitute a public health strategy (9, 10).

Despite being recognized for their functional properties, the technological incorporation of polyphenolic compounds in food or pharmaceutical formulations could be more feasible due to pronounced molecular instability (9, 11). They are sensitive compounds to environmental conditions such as temperature, changes in pH ranges, and luminosity (12, 13). Polyphenolic compounds have limited stability in human gastrointestinal ambient, such as intestinal pH, enzyme action, and intestinal microbiota, reducing the absorption of intact structures and bioavailability and significantly affecting functional activity (14, 15). Thus, the nanoencapsulation of these natural compounds can be an alternative to enable technological inclusion in different food matrices (16).

Natural polysaccharides extracted from fruit peel can be used as resistant biomaterials to form nanostructures to encapsulate chemically unstable compounds such as polyphenols (17). In recent years, complex polysaccharides such as pectins have been studied for this purpose (18, 19). They are low-toxic compounds, biodegradable, biocompatible, and widely applicable in food products. Therefore, polysaccharides extracted from food residues are composed of a sustainable source for elaborating efficient nanosystems to overcome the (20, 21) physical–chemical instability of some bioactive compounds (22, 23). The nanoencapsulation process maintains the integrated structure of stable bioactive compounds that is better biologically utilized than free compounds and enables specialized application in the food and pharmaceutical industries (24).

The scientific literature has reported that polyphenolic compounds have functionalities and bioactivities (anti-aging, anti-inflammatory, antioxidant, and antiproliferative) that effectively promote human health (9). Moreover, there is an emerging global need for sustainable actions for using residues from the food industries (25). Therefore, this review summarizes current knowledge about the properties of polyphenols from by-products and discusses the implications of nanoencapsulation with polysaccharides extracted from by-products to stabilize polyphenols. The basic principles of the full use of food residues as primary sources of phenolic compounds and the advantages of nanoencapsulation techniques applied to protect these phytochemicals, thus creating nano-fortified foods and supplements. Polysaccharides, such as pectins, are biomaterials indicated for this purpose, and together with the main techniques, they will be discussed. The explanation of aspects related to the extraction of compounds from by-products and the inclusion of food products through nanoencapsulation is essential to instigate future research with impacts on the environment and, simultaneously, stimulate the consumption of bioactive for health promotion.

## 2. The molecular structure and role of polyphenolic compounds for human health

Polyphenolic compounds are extensive and heterogeneous phytochemicals in plant-based foods (26, 27). They are among plant tissues’ most important secondary metabolites, such as flowers, seeds, peels, roots, and edible parts. They are widely distributed in various food sources, such as wine, tea, coffee, cereal grains, and vegetables, such as multiple fruits (28–30).

Polyphenols have a wide diversity of structures, changing their functional properties according to their molecular composition. They are constituted by aromatic rings (benzene) with attached hydroxyl groups, organic acids, sugars (mono-, di-, or oligosaccharides), and acylated sugars that are conjugated to primary phenolics structure with many hydrophilic groups (27). They are composed of two phenyl groups linked by a three-carbon bridge, with different degrees of oxidation and unsaturation of the three-carbon segment, and various sugar units associated in various positions of the polyphenol structure – or associated with organic acids or both – can be attached to the hydroxyl groups of flavonoids (31). Phenolic acids consist of a single phenyl ring, and the molecular structure of these compounds can affect their absorption and, consequently, their functional properties. Despite attributing positive effects to the metabolites, the bioavailability and activity of polyphenolic compounds are directly linked to their intact structure. Some aspects can affect molecular stability, such as the interactions with other food constituents and factors intrinsic to human digestion, such as intestinal pH and microbiota (32, 33). The functional properties (antioxidants) protect cells against oxidative damage. However, these compounds’ biological activity directly depends on the structural and glycosylation patterns (34).

**Figure 1:**
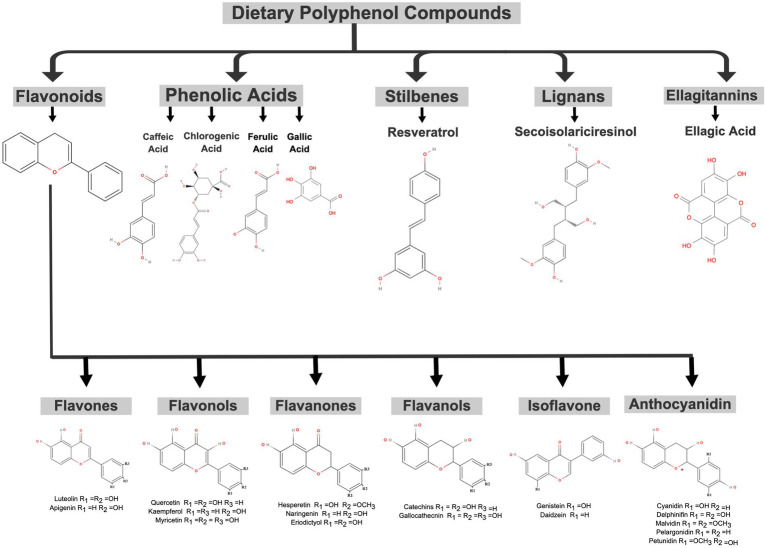
Chemical structures of polyphenolic compounds in foods. The basic structure forms of the main groups are depicted: flavonoids, phenolic acids, stilbenes, lignans, and ellagitannin and their subclasses. The figure was created with MolView (https://molview.org) and Mind the Graph (https://mindthegraph.com) (accessed on 10 April 2023).

Polyphenolic compounds are present in the human diet, and their regular consumption is highly recommended (34, 35). The sources of dietary polyphenols in nature are vast, and they can be found in various plant-based foods, including cereals, teas, chocolates, vegetables (such as broccoli, onions, and cabbage), and fruits (such as grapes, pears, apples, cherries, and others). In berries, the content can vary from 200–300 mg of polyphenols for each 100 g of total fresh weight (34, 36–38). Dietary polyphenols are divided into subclasses according to their chemical structures, from simple ones (single aromatic ring), such as hydroxycinnamic acids, to complex structures, such as ellagitannins (37). The main classes found in plants are flavonoids, phenolic acids, lignans, and stilbenes. Flavonoids are considered the most abundant polyphenolic compounds in food. The different structures can be subdivided into six main subclasses: flavonols, flavones, flavanones, flavanols, isoflavones, and anthocyanidins (39). The basic structure of flavonoids comprises three aromatic rings linked together and different radicals attached to the primary structure. In addition, the position and number of hydroxyl and glycosylation groups also differ in other flavonoids. Resveratrol, a subclass of stilbenes, and some phenolic acids, such as caffeic acid, chlorogenic acid, and ferulic acid, are some examples of polyphenols (40–43). The chemical structures of dietary polyphenol groups in foods are shown in Figure 1.

Flavonoids are the predominant polyphenols in the human diet, and many foods are sources of these phytochemical classes. Anthocyanins (glycosylated anthocyanidins) are present in foods such as blackberries, strawberries, grapes, red cabbage, and blueberries. They are responsible for various plant species’ blue, purple, pink, and red colors. Quercetin is the main flavonoid in onions, tea, and apples. Citrus fruits are the primary sources of hesperidin, and soy is an essential source of genistein and daidzein. Tea, cocoa, chocolate, and beans are sources of other flavonoids. In turn, flavanones are primarily present in oranges, grapefruits, and lemon; flavonols in onion, broccoli, apple, berries, bean, and red wine and flavones in capsicum pepper (43–48). Recent studies suggest that appropriate combinations of polyphenols (quantity and proportion) can increase their bioactivity (49).

The benefits are mainly due to the antioxidant action – but not the only one – as they are considered a natural reactive oxygen species reducer and can donate hydrogen atoms and electrons (32, 50, 51). They are potent antioxidants and can prevent oxidative damage (reactions mediated by free radicals) of biomolecules such as proteins, nucleic acids, polyunsaturated lipids, and sugars (52, 53). The structure–activity relationship of polyphenolic compounds interferes with the antioxidant activities, such as the number and positions of the hydroxyl group (–OH), the presence of a double bond (C2 = C3), glycosylation and the presence of radicals attached to the primary structure (33, 54, 55). The hydroxyl groups donate hydrogens and electrons to stabilize the radicals. The ring with two hydroxyl groups (–OH) indicates a high antioxidant effect. Also, the number of hydroxyls is related to the increase in the hydrophilicity of the molecule (54). The double bond in molecular structure (C2 = C3) with a 4-carbonyl group provides planarity, electron expansion, and displacement between adjacent rings, altering the dissociation constant of the hydroxyl groups and favoring the radical stabilization, thus increasing the antioxidant activity (36, 54). The presence of glucose also affects the antioxidant activity, while the *C*-glucoside form demonstrated greater antioxidant capacity when compared to *O*-glycoside due to planarity, methylation, and electron displacement (54, 56). The mechanisms of antioxidant action can occur through two distinct pathways (57). In the first mechanism, the free radical (R) removes a hydrogen atom from the antioxidant (ArOH) (Scheme 1). The second mechanism involves donating an electron to the free radical, which becomes a cation radical (Scheme 2) (53, 57).

1. R^.^ + ArOH → RH + ARO˙
2. R^.^ + ArOH → RH^−^ + ARO˙ ^+^

Plant proteins derived from residues are considered ideal biomaterials to nanoencapsulate phenolic compounds for protection and controlled delivery (58–61). They can be used alone or combined with polysaccharides (17, 62). Phenolic compounds and proteins can interact and form complexes (through covalent and non-covalent bonds) through hydrophobic, electrostatic, *van der Waals* forces, and hydrogen bonds. Interaction between polyphenolic compounds and proteins can change the conformation of the protein structure, lead to folding or unfolding, forming insoluble or soluble complexes, affecting their nutritional and functional properties and their bioactivities that could generate beneficial effects on human health (63, 64). On the other hand, molecular interactions between proteins and polyphenolic compounds can favor molecular stability and modify polyphenols’ physical and chemical properties, thus improving intestinal release and absorption (65).

Polyphenolic compounds may synergistically affect cellular signaling to increase the expression of molecules, including receptor proteins, enzymes, cofactors, and regulators. Furthermore, these compounds can also regulate different signaling, such as activating convergent mitochondrial signaling pathways (66–68). Polyphenolic compounds can regulate intracellular signaling pathways, targeting intracellular enzymes, transcription factors, receptors, and other functional proteins regulation (64, 65, 69–71). Due to these mechanisms, polyphenolic compounds have anti-inflammatory action, anti-aging, antibacterial, cardio-protective, neuro-protective, and anticancer effects (5, 49, 72).

Another aspect is the ability of polyphenolic compounds positively impact human health due to systemic effects (73, 74). Polyphenols can induce microbiota homeostasis, maintaining intestinal barrier integrity (75, 76). They can modify the composition of the microbiota by increasing the ratio of beneficial bacteria to pathogenic ones and promoting immunomodulatory and prebiotic effects (77, 78). At the same time, intestinal microbiota can biotransform some of the polyphenols into more active metabolites. The literature has reported that, although not fully elucidated, changes in the microbiota composition affect the neuronal and endocrine systems. The microbiota-gut-brain is an essential neuroendocrine system that bidirectionally regulates the development of some neural disorders. Thus, the positive effect of phenolic compounds in the gut is initially due to the modulation of the local microbiota, extending to systemic impacts (79–81).

Several studies support that consuming these phytochemicals is associated with health benefits. Particularly, anthocyanin consumption has been associated with the prevention of type 2 diabetes mellitus (82), the prevention of metabolic diseases and obesity (32, 83), the cardio-protective potential, the protection of the gastrointestinal tract from high fat diet-induced alterations in redox signaling, barrier integrity and dysbiosis (84), the anticancer effects (85), and the decreased oxidative stress and inflammation (86), and many other positive effects on human health (3).

The biological properties of polyphenols indicate the numerous applications for the chemical, pharmaceutical, and food industries. Developing epidemiological strategies to enrich foods with these compounds may constitute a public health strategy to reduce the risks of developing diseases like the ones mentioned above. In addition, dietary supplements enriched with polyphenols may be an effective way to increase consumption in the population, with favorable long-term results (16, 87, 88).

## 3. Molecular stability of polyphenolic compounds through nanoencapsulation systems

Molecular instability limits the inclusion of polyphenols in foods and reduces the biological effects due to low absorption. Limited bioaccessibility and bioavailability are challenges to be overcome through innovative technologies (17, 89–92). The combination of polyphenolic compounds with other compounds (biomacromolecules) in nanostructures can protect them from factors intrinsic to human digestion (intestinal pH, action of digestive enzymes, and intestinal bacteria) (11, 16, 93). Thus, the wide use of polyphenols is limited due to their poor stability (maintenance of molecular integrity) under gastrointestinal factors, which significantly impact the effectiveness delivered to the target tissues to perform their biological function (92, 94).

The technological use by the food and pharmaceutical industry is impracticable since, during the processing and storage, these compounds are susceptible to several factors, limiting their application (59, 95). Environmental conditions, such as temperature, light, oxygen, pH, enzymes, and the presence of other food compounds, are considered the main factors that degrade these bioactive compounds. Processing, storage, and digestion conditions can cause chemical and structural changes and instability (96, 97). Also, the temperature in the food industry is essential to preserve microbiologically, and to improve texture and flavor characteristics, impairing the addition of these antioxidants before this step (98). Current research points to nanoencapsulation as a potentially efficient approach for protecting polyphenolic compounds from adverse environments, providing stability to maintain the properties, thus enabling technological use (99–101). In addition, scientific literature has reported a significant loss of properties and functionality of polyphenolic compounds when exposed to environmental factors, processing, and human digestion (89). The high temperature and increase in pH values in the period of storage and food processing by the industry are the main factors responsible for polyphenols degradation (59). Molecules such as anthocyanins are susceptible to the environment and easily degraded, limiting their application as a natural dye. Molecular instability impacts color, decreasing the intensity and affecting other sensory characteristics of the food (102, 103).

When ingested, polyphenolic compounds are extensively degraded and biotransformed by the action of digestive enzymes, intestinal pH, and intestinal bacteria (76). Studies have shown that only small amounts of intact molecules are available for absorption after oral ingestion. In the gastrointestinal tract, degraded polyphenols have less absorption, high excretion, decreasing antioxidant activity, and consequent lower biological activity than intact molecules (104, 105). Many polyphenols reach the colon and are biotransformed by the local microbiota, resulting in different metabolites that can be absorbed or excreted (76, 106). In the intestine, polyphenolic compounds undergo successive phases of biotransformation and degradation. In the large intestine, several reactions occur with polyphenols, such as deglycosylation, dihydroxylation, *α*- and *β*-oxidation, dehydrogenation, demethylation, decarboxylation, C-ring fission, and cleavage to lower molecular weight (36, 107). Additionally, the molecular composition, bound radicals, size, charge, hydroxylation, glycosylation, acylation and pigmentation, possible matrix effect, and presence of specific transporters are some factors that influence structural stability, antioxidant capacity and absorption of polyphenols in the human organism (108, 109).

The low bioavailability of these compounds results in decreased bioefficacy and decreased health effects. It hinders technological application, which limits all the health benefits attributed to *in vitro* and *in vivo* studies and restricts specialized applications (108, 110). Bioavailability can vary between different polyphenol compounds, but bioavailability is generally low for most of these polyphenols due to molecular instability being the determinant factor. The biotransformation in the gastrointestinal tract is basically due to two main factors. The first is specific to the molecular structure of the phenolic compounds because, according to the form (scaffold), it can facilitate metabolization by the human intestinal enzymes and the intestinal microbiota. The second factor responsible for the low biological use concerns the diversity of the colonization of the intestinal microbiota. Some structural alterations of polyphenols (such as deglycosylation) occur by more generic groups of bacteria, while others arise by specific bacterial genera (111–113).

There are several technologies of nanoencapsulation to maintain molecular stability, reduce the degradation of polyphenols, and develop an efficient delivery system (100, 114, 115). Nano-functional plant-based foods have aroused growing interest from researchers to investigate the mechanisms for metabolic diseases, such as prevention and adjuvant treatment, including the disorders mentioned in item 2 (62, 116). Nanotechnology is considered a new frontier for improving food quality in the food sector, resulting in increased nutritional value and food safety. For the health area, nanoscience corresponds to the targeted delivery of nutrients and bioactive compounds in the body, thus possibly impacting disease decrease (117). In addition to the search for applied technologies to maintain molecular integrity/stability, there has been a need to identify alternative sources for extracting polyphenolic compounds. In recent years, sustainable sources have been explored in line with current demands for environmental preservation and the development of nano-functional foods (118, 119).

## 4. The use of waste from food industries: possible benefits for human health, and prospecting the reduction of impacts on the environment

Recent research indicates that food systems significantly affect climate change (120). It is estimated that in the subsequent years, the impact on the environment caused by the inefficient use of natural resources, concurrent with the exponential growth of the world population, will result in profound social consequences (118, 121). The production of residues from the food industry significantly impacts the environment, constituting approximately one-tenth of food systems emissions (122, 123). This complex panorama indicates a current urgency for actions aimed at the sustainable use of natural sources, such as reducing waste and optimizing the use of by-products from the food industry.

Food losses and waste are part of the production chain, starting at the harvest, passing through the industrial processing stage, and throughout distribution. According to data from the FAO (United States Food and Agriculture Organization) around the world, approximately 14% of the world’s food – about US$ 680 billion in developed countries and US$ 310 billion in developing countries – is lost annually (124, 125). The agro-food system supply chain produces significant by-products (118). The considerable amount of food discards and biomass generates an accumulation in nature, increasing environmental and economic impacts (120).

Regardless of the considerable progress in industrial residue management in the world in the last decades, the remaining natural by-products still need to be used to their maximum potential, indicating a critical and current problem (25). The need for public policies to reduce food losses in the production chain has recently been discussed, as well as the impact on the sustainable use of environmental resources for the economy of countries and on social issues related to food and nutrition security (124, 126). The United Nations (UN) has been encouraging actions to achieve sustainable development goals, which include specific proposals such as “Zero Hunger,” “Good Health and Well-Being,” “Responsible Production and Consumption,” and “Climate Action.” Innovative strategies that culminate in these objectives must be instigated, mainly those that seek the development of concrete actions for the full use of shared natural resources and direct continuous efforts to reduce food waste while promoting the health of populations (120, 123, 127, 128).

Although several technological approaches aim to reduce the environmental impacts of food industries, some challenges remain open (129, 130). Globally, millions of tons of food waste from vegetables are generated annually, mainly from processing fruits and grains (131). Food sub-products constitute a huge extractive source of nutrients such as vitamins, minerals, and macromolecules (proteins and polysaccharides) (121, 132). In addition, vegetable residues, mainly from fruit peel, can extract non-nutrients, such as bioactive phytochemicals. The wide diversity of these compounds includes several groups, comprehending carotenoids, prebiotics and dietary fibers, pigments, and phenolic compounds – most of them with remarkable beneficial bioactivity (26, 133). Sustainability actions are being planned, such as the rational use of raw food materials and the correct disposal of by-products (121). In this sense, the technological use of food waste can be an alternative to reduce environmental impacts with positive economic consequences (127). In addition, the extraction methods must also be carefully selected, with environmentally friendly techniques, and exclude potentially toxic compounds (119, 134, 135).

Pereira et al. (121) indicated that adding value to the wasted parts of food can be a strategy to mitigate food shortages in the future. The use of peels and seeds, in addition to being nutritionally adequate, can have positive environmental impacts in the long term. The use of by-products from the food industry as alternative sources for the extraction of polyphenolic compounds and the development of supplements can be an action to provide functional products to the population while attributing value to the waste (131). The use of vegetable peels for the characterization and extraction of polyphenols and the destination for the enrichment of products can be a promising option for this purpose (119, 124).

Some of the extracted phenolic compounds (e.g., anthocyanins) can be used as pigments and natural dyes, replacing synthetic ones with vast industrial applications and commercial interest (103). Due to the beneficial health properties already discussed, they can be used as an additive to nano-foods due to their properties, and for the development of new functional products/ingredients and supplements for use as adjuvant treatments in different physiological processes (116, 136, 137), upcycling by-products into biofunctional components (26, 119).

Suleria et al. (26) indicated that fruit peels have diverse phytochemicals, including phenolic compounds. The levels of the bioactive compounds were identified through a comprehensive screening and characterization/quantification by HPLC and LC–MS/MS in different fruit peels. The antioxidant content was attested and the potential use for extracting phenolic compounds in the peel of twenty other fresh and ripe fruits was proposed, such as apple, apricot, avocado, banana, custard apple, dragon fruit, grapefruit, kiwifruit, lime, mango, melon, nectarine, orange, papaya, passionfruit, peach, pear, pineapple, plum, and pomegranate.

The sub-product sources for polyphenolic compounds are varied and widely available and indicate a potential unexplored source for extraction. Table 1 shows some of the residual sources rich in phenolic compounds, and different studies (*in vitro* and *in vivo*) are described to indicate the biological effects.

**Table 1:** According to the group and sub-group, polyphenolic compounds in by-products of the food industry, and the main effects are reported in studies *in vitro* e *in vivo*.

Chemical class	Chemical sub-class	Polyphenolic compounds	Molecular formula	By-products/peel: possible source for extraction	Reported effects	Reference
Phenolic acid	Hydroxybenzoic acids	Gallic acid 4*-O-*glucoside	C_13_H_16_O_10_	Apple, apricot, grapefruit, mango, orange, passionfruit, pear, pineapple, plum, pomegranate, jaboticaba	Benefits for the cardiovascular system (hypertension, atherosclerosis, and dyslipidemia).	(138)
		Protocatechuic acid 4*-O-*glucoside	C_13_H_16_O_9_	Apple, apricot, banana, grapefruit, kiwifruit, mango, orange, passionfruit, pear, pineapple, plum, pomegranate, papaya, coffee	Beneficial effect of regulating blood lipids Higher antioxidant capacity Attenuate changes induced by high-fat diet in rats	(139, 140)
		Vanillic acid 4-sulfate	C_8_H_8_O_7_S	Mango, pear, kiwifruit	Action on intestinal barrier and urinary epithelium	(141)
		Ellagic acid arabinoside	C_19_H_14_O_12_	Orange	Antimicrobial properties against a wide range of microbial pathogens Neuroprotective potential	(142–144)
	Hydroxycinnamic acids	Caffeoyl tartaric acid	C_13_H_12_O_9_	Plum, mango, orange, passionfruit	Antibacterial potential and Antioxidant Capacity	(145)
		Isoferulic acid 3-sulfate	C_10_H_10_O_7_S	Plum	Antioxidant potential	(146)
		Ferulic acid 4-O-glucoside	C_16_H_20_O_9_	Apricot, kiwifruit, mango, nectarine, pineapple, plum, pomegranate, avocado, custard apple, papaya	Antioxidant and potential pharmaceutical properties	(147)
		Caffeic acid 3*-O-*glucuronide	C_15_H_16_O_10_	Custard apple, grapefruit, orange, kiwifruit, pineapple, dragon fruit	Antioxidant properties	(148)
	Hydroxy phenylacetic acids	3,4-Dihydroxyphenylacetic acid	C_8_H_8_O_4_	Apple, apricot, custard apple, grapefruit, mango, melon, nectarine, peach, orange, pear, passionfruit, plum, pomegranate, avocado	Relax arteries *ex vivo* and decrease blood pressure *in vivo*	(149)
	Hydroxy phenyl propanoic acids	Dihydroferulic acid 4-sulfate	C_10_H_12_O_7_S	Avocado	Antioxidant potential	(150)
Flavonoids	Flavonols	(−)-Epigallocatechin	C_15_H_14_O_7_	Avocado	Cellular targets and inhibits cancer cell proliferation by inducing apoptosis and cell cycle arrest.	(151)
		Cinnamtannin A2	C_6_0H_50_O_24_	Custard apple, kiwifruit, plum, avocado, dragon fruit, pine bark, grape	Improve cognitive function	(152)
		(+)-Catechin 3*-O-*gallate	C_22_H_18_O_10_	Kiwifruit, pear, avocado	Chemoprotective mechanism reduce oxidative stress	(153, 154)
	Flavones	Apigenin 6,8-di-C-glucoside	C_27_H_30_O_15_	Apple, apricot, grapefruit, kiwifruit, orange, papaya, passionfruit, peach, plum, lime, melon	Antioxidant activity	(155)
	Flavanones	Narirutin	C_27_H_32_O_14_	Apple, nectarine, dragon fruit, lime	Antioxidant and anti-inflammatory activities	(156)
		Hesperidin	C_28_H_34_O_15_	Lime	Inhibitory effect against obesity diseases regulates lipid metabolism, glucose metabolism, and antioxidant action. Neuroprotective effect	(157, 158)	
	Flavonols	Myricetin 3-O-rutinoside	C_27_H_30_O_17_	Lime, mango, nectarine, peach, passion fruit, avocado	- Antioxidant, anticancer, antidiabetic, and anti-inflammatory activities - Anticancer effects	(159, 160)	
		Quercetin 3’-O-glucuronide	C_21_H_18_O_13_	Lime, orange, pomegranate, kiwifruit	Effective in ameliorating endothelial insulin resistance by inhibiting reactive oxygen species-associated inflammation. Promotes the proliferation and migration of neural stem cells	(161, 162)	
		Myricetin 3-O-galactoside	C_21_H_20_O_13_	Banana, orange, pomegranate	- Antioxidant, anticancer, antidiabetic, and anti-inflammatory activities - Anticancer effects	(159, 160)	
		Kaempferol 3,7-O-diglucoside	C_27_H_30_O_16_	Apple, apricot, nectarine, peach, orange, passion fruit, plum, lime, papaya, beans, broccoli, cabbage, gooseberries, grapes, kale, strawberries, tomatoes, citrus fruits, Brussel sprouts, grapefruit	-Prevention and treatment of inflammatory diseases. - Chronic inflammation-induced diseases, anticancer, and anti-obesity. - Antioxidant properties	(163–166)	
	Dihydrochalcones	3-Hydroxyphloretin 2’-O-xylosyl-glucoside	C_26_H_32_O_15_	Apple, mango, pear, pineapple	Antioxidant properties	(167, 168)	
	Dihydroflavonols	Dihydroquercetin	C_15_H_12_O_7_	Custard apple, kiwifruit, mango, peach, pear, papaya	Antioxidant properties	(168, 169)	
	Anthocyanins	Delphinidin 3-O-glucoside	C_21_H_21_O_12_	Custard apple, avocado, kiwifruit, papaya, pomegranate, jaboticaba, grape	Activate the immune response in the tumor microenvironment (human colorectal cancer cells) and induce cancer cell death *in vitro.* Inhibits angiogenesis via VEGFR- 2* down-regulation and migration through actin disruption. Antiproliferative effect on several types of solid tumor cancer cells.	(170–172)	
		Cyanidin 3,5-O-diglucoside	C_27_H_31_O_16_	Avocado, custard apple, kiwifruit, lime, papaya, peach, dragon fruit, blackberry, grape, strawberry, jaboticaba	Activate the immune response in the tumor microenvironment (human colorectal cancer cells) and induce cancer cell death *in vitro*. Antioxidant activity	(170, 173)	
		Pelargonidin 3-O-rutinoside	C_27_H_31_O_14_	Lime	Hyperglycemic control Antioxidant properties	(174, 175)
Lignans	Secoisolariciresinol-sesquilignan		C3_0_H_38_O_10_	Avocado, custard apple	Antioxidant capacity	(176, 177)
Stilbenes	Resveratrol		C_14_H_12_O_3_	Custard fruit, avocado, dragon fruit, blackberry, grape	Regulates immunity by interfering with immune cell regulation and proinflammatory cytokines. Potentially improve the therapeutic outcome: diabetes mellitus, obesity, colorectal cancer, breast cancer, multiple myeloma, metabolic syndrome, hypertension, Alzheimer’s disease, cardiovascular disease, and rhinopharyngitis	(178, 179)

* VEGFR-2: Vascular endothelial growth factor receptor-2.

**Figure 2:**
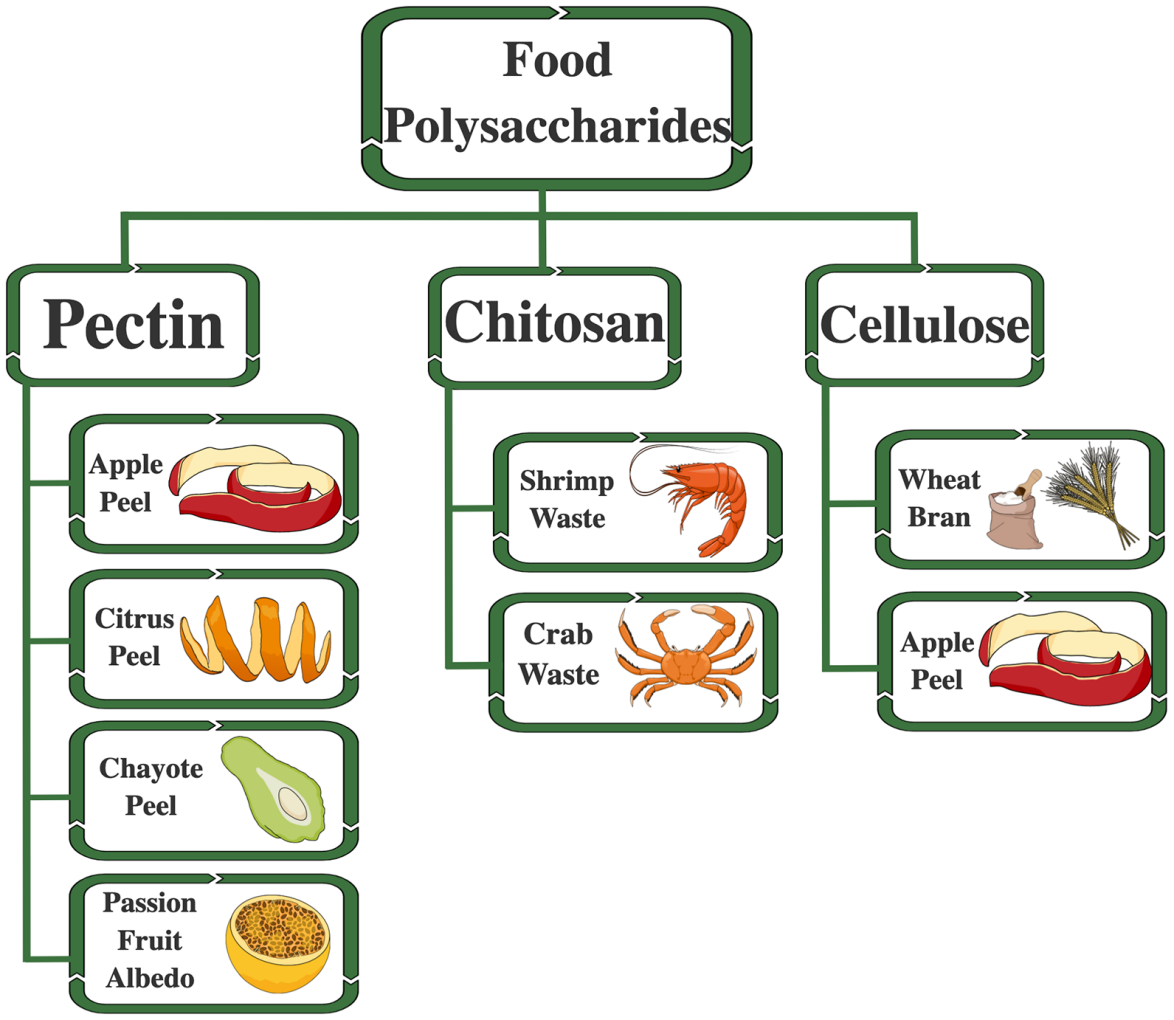
Polysaccharides are found in by-products of the food industry, the primary sources for extraction available. Polysaccharides are considered potential biomaterials to form nanocapsules for the entrapment of chemically unstable compounds. The figure was created by Mind the Graph (https://mindthegraph.com) (accessed on 10 April 2023).

Most of the macromolecules extracted in abundance from by-products can be considered sustainable, with high quantities for extraction (118, 131). Non-starch polysaccharides, such as pectins, chitosan, and cellulose, are abundant in industrial food waste and with well-established extraction and isolation methods (134, 180) (Figure 2). These biomaterials can be used to form nanostructures for encapsulating compounds highly sensitive to environmental and biological factors (181).

Food polysaccharides are used in food technology applications as physical modifiers and gelling agents for foods and drug formulations. Especially the non-starch polysaccharides are gastro-resistant and are used to compose drug delivery systems (33, 182). The various new functionalities and biological effects are being extensively explored (183), and nanotechnology is a new possibility for applying these biomaterials, such as pectins (62). Pectins are complex polysaccharides of plant origin and are widely distributed in nature, with excellent application in nanotechnology for food and pharmaceutical industries (184).

Pectins are abundant in wasted food because they are constituents of the plant cell wall and form a primary layer that surrounds and supports plant cells. They are in various vegetables, mainly citrus peel and albedo (orange, lemon, lime, and pomelo), apple peel, chayote peel, and passion fruit albedo. All these by-products from fruits are considered adequate sources for the extraction of pectin (Figure 2) (185–187). The industrial processing of citrus fruits to produce juices is one of the primary sources for obtaining pectin, and several agro-industrial residues are being investigated as potential extractive sources. The peel and albedo of remaining fruits are ~50% of the total fruit, with 25 to 30% (dry weight of citrus peel) composed of pectin (134, 188–190). Other alternatives sources can be used to obtain pectins, such as sugar beet and sunflower seed head (191), tomato waste (192), cocoa husks (193), grapefruit peel, pomegranate peel, passion fruit peel, mango peel, banana peel, and kiwi fruit pomace (194–199).

The extraction, purification, and fractionation of pectins from alternative sources can be considered sustainable. The physical, chemical, rheological, and functional properties of pectin indicate the potential for technological application in several segments since pectin is stable in the digestive tract, with excellent mechanical resistance, biodegradability, and biocompatibility (188). Therefore, pectin is a promising biomaterial to compose nanostructure systems to protect chemically unstable compounds, such as phenolic compounds (8, 23, 200). Other characteristics and applicability of nanostructured pectins will be discussed later in this article.

## 5. Biomaterial for elaborating nanostructure systems for encapsulating polyphenolic compounds from food residues

To increase the economic value of the compounds extracted from losses/residues and by-products of the food industry, phenolic compounds and pectins can be applied in nanotechnology (16). Using vegetable peels to extract these two natural compounds represents the valorization of the agro-food losses and waste from the food industry while constituting a strategy to produce new products with added economic and nutritional value (118). Using nanoencapsulated polyphenols as a natural and potent source of antioxidants in the various segments of the food and pharmaceutical industries is considered an innovative approach (59).

Over the last few decades, various technologies were developed to encapsulate and consequently protect polyphenols, such as microencapsulation (201, 202). Although effective, technology at nanometric scales (sizes <1,000 nm), such as nanoemulsion, nanoliposomes, nanoparticles, and nanogel, is indicated to increase stability, bioaccessibility, controlled delivery, and bioavailability of phenolic compounds with numerous application possibilities (7, 114, 203, 204). Notably, there is a significant difference between the bioavailability of free and nanoencapsulated polyphenols (100, 116).

The nanoencapsulation techniques physically and chemically stabilize the compounds, promoting better absorption of intact forms, thus improving the bioavailability. Nanoencapsulation can also increase storage stability, enabling its use as a food ingredient (16, 100, 115, 205). The nanotechnology process can also enhance some sensorial characteristics, such as the taste and astringency of some polyphenols (16, 115). Moreover, different nanoencapsulation systems can be used to optimize the controlled delivery of polyphenolic compounds (93, 114). Two distinct approaches are reported for the formation of nanostructures: (I) top-down and (II) bottom-up. The top-down approach aims to reduce the size of structured material to a nanometer scale through external disruptive mechanical forces using precise tools/equipment. On the other hand, the bottom-up approach involves using molecules by self-organization/self-assembly (62, 200). Factors such as proportion and concentration between compounds (encapsulated and encapsulating), temperature, pH, agitation, and ionic strength must be carefully controlled to form nanocapsules (206, 207).

Nanoencapsulation means a scientific and technological effort that can reduce food waste by upcycling by-products to generate value-added products, highly specialized and intended for broad application in the food industry (food, health, pharmaceutical, and cosmetic areas). Nanotechnology is an alternative to value this industrial waste and can contribute to global sustainability actions (16, 119). Different materials such as fruit peels as sources of polysaccharides to form nanostructures – for improving the stability of labile compounds – indicates an innovative possibility for introducing new resources, such as expanded technological use of phytochemicals and their controlled release in the specific tissues/organs. The impacts on the use of by-products of the food industry through the application of nanotechnology polysaccharide-based constitute opportunities and innovations (11, 16, 17, 116, 131).

Several scientific investigations are directed toward identifying methods to retain and improve the polyphenolic content and delivery to the human body. The antioxidant activity of polyphenolic compounds extracted from coffee grounds was preserved through encapsulation based on polysaccharides (maltodextrin, gum Arabic, 1:1 ratio) and with techniques such as lyophilization and spray-drying (30). The study done by Kasote et al. (208) reviewed several studies that used different methodologies for stabilizing polyphenolic compounds and their inclusion in cereals. Nanoencapsulation was cited as a promising technology and as one of the options for developing functional foods with bioavailability improvement (16, 59, 116).

Polysaccharides, such as pectin, are important biomolecules to be used as biomaterials for sectors of the food and pharmaceutical industries. These natural polysaccharides’ properties, bioactivities, and versatility can be applied in nanotechnology (209). Furthermore, pectins are found in many renewable sources and food by-products due to their wide distribution in nature (180). Nanoencapsulation based on pectin has attracted attention due to favorable properties, such as large specific surface area, excellent stability, good biocompatibility, improved permeability and retention time of the encapsulated compound, uncomplicated design and preparation, structural flexibility and preferred characteristics of controlled release in the intestine, and delivery to specific physiological sites (210–212). In addition, the nanoencapsulation process contributes to improved antioxidant properties and prebiotic effects (213).

Food polysaccharides can undergo reversible self-assembly and respond to stimuli such as ionotropic gelation. Besides, they interact with other molecules through self-assembly, generating structures on a nanometric scale (214, 215). The process can be performed without adding surfactants, emulsifiers, or other potentially unsafe products for human ingestion and the environment (62, 215). Scientific literature has reported that different approaches and varied techniques are used to prepare nanostructured polysaccharides, proteins, and lipids. They can be used alone or with other food biomacromolecules, forming nanocomplexes to encapsulate chemically unstable compounds (16, 216). For example, some coating materials are indicated for anthocyanin’s protection (24). Natural polysaccharides are indicated due to their high nutritional value and potential functional health properties. In this sense, some studies demonstrated the pectin-based nanoencapsulation of anthocyanins resulting in physical–chemical protection, enabling more excellent stability and color maintenance and aiming at an increase in intestinal absorption (18, 184, 217). Polyphenolic compounds were encapsulated in pectin-based nanoparticles (isolated or combined with proteins) with excellent results and repeatability (205, 218).

As depicted above, food biopolymers (polysaccharides and proteins) are suitable for wall material encapsulation (219). Several studies successfully used pectin to encapsulate phenolic compounds, isolated or combined with other polysaccharides (chitosan and cellulose) and proteins (lysozyme and whey protein) (18, 205, 217, 218, 220, 221). Table 2 shows the most recent studies that used different biomaterials to nanoencapsulation polyphenolic compounds (extracted from other sources), indicating the versatility of combinations between biomacromolecules and different purposes and applications. The various research highlights the main objectives for nanoencapsulation of phenolic compounds, which are maintaining the stability, the color, and the application in food matrices for resistance during the processing. In addition to industrial applicability, increased bioaccessibility, antioxidant capacity, and bioavailability are identified as the biological factors that motivate the development of nanocapsules.

**Table 2:** Nanoencapsulation of polyphenolic compounds polysaccharide and protein-based nanostructures.

Encapsulating material	Phenolic compound	Method	Size (nm)	Zeta potential (mV)	Encapsulation efficiency (%)	Purpose	Reference
Pectin - Cellulose	A blend of phenolic compounds extracted from pomegranate peel	Nanoemulsion	~200	–	~20	Highest antimicrobial activity	(220)
Carboxymethyl chitosan	Epigallocatechin gallate	Ionic cross-linking	400	+36.6	75	Increase antitumor activity	(222)
Pectin – WPC	Olive leaf phenolic compounds	Nanoemulsion	347.7	–	72.73 to 96.64	Increased antioxidant properties and release rate	(205)
Pectin – WPI	Anthocyanin	Self-assembly	200	−36	55	Improve Stability	(184)
Carboxymethyl chitosan	Anthocyanin	Ionic interaction	219.53	–	63.15	Promote stability and inhibit degradation in the gastrointestinal tract	(224)
Chitosan	Catechins	Polyanion-gelation	169.0 to 201.4	~ +30	24 to 53	Targeted delivery system	(225)
Chitosan and β-lactoglobulin	Epigallocatechin gallate	Ionic gelation	100 to 500	+10 to +35	~ 60	To release epigallocatechin gallate in the gastrointestinal tract	(226)
Chitosan-Alginate	Anthocyanins	Ionic pre-gelation and polyelectrolyte complex formation	358.5 to 635.9	–	56.87 to 68.9	Maintenance of antioxidant activity	(227)
Chitosan	Anthocyanin	Ionic gelation	160 to 1,093	–	–	To stabilize the color and the antioxidant activity	(228)
Chitosan - β-Lactoglobulin	Anthocyanin	Ionic gelation	91,71	–	69.33	To improve stability and bioavailability	(223)
Soybean insoluble dietary fiber	Anthocyanin (Malvidin-3-O-glucoside)	Emulsification	300	–	–	Storage stability and protection of color	(229)
Chitosan	Anthocyanin	Ionotropic gelation	274 to 455	−5.04 to −35.4	70	To improve in vivo antioxidant potential	(230)
Pectin	Resveratrol	Antisolvent precipitation and electrostatic deposition	120	−30	–	Stability, Bioaccessibility, and Antioxidant Capacity Maintenance	(218)
Pectin–Chitosan	Anthocyanins	Self-assembly	100–300	–	66.68	Molecular Stability and Control Released	(215)
Pectin–Lysozyme	Anthocyanins	Self-assembly	198.5	−26	73	Molecular Stability	(18)
Chitosan–Pectin	Anthocyanin	Polyelectrolyte complex	–	+ 37 to +55.5	60	Color protection and application in food packaging	(221)
Pectin-Chitosan	Anthocyanin	Nanoliposomes	64 to 352	−30 to +21	28.54 to 61.17	To investigate the protective effect of hepatocytes injury in L02 cells	(231)
Hohenbuehelia serotina polysaccharides	Quercetin	Self-assembly	360	−38.8	21.41 to 52.28	Maintenance of stability and its anti-proliferative activities during *in vitro* gastrointestinal digestion	(232)
Chitosan - β-Lactoglobulin	Anthocyanin	Ionic gelation	580.4	+49.6	77.4	Storage stability and oxidant stability during *in vitro* simulated digestion	(233)
Zein–Carboxymethyl cellulose	Quercetin and Resveratrol	Antisolvent Precipitation	217	−33.6 to −45.6	25,1	Thermal stability and orally administered	(234)

WPI, Whey Protein Isolate; WPC, Whey Protein Concentrate.

As shown in Table 2, polysaccharides – isolated or bound to proteins – are predominantly used for the nanoencapsulation of anthocyanins, epigallocatechin gallate, catechins, and resveratrol. The nanoencapsulation of these polyphenolic compounds was developed to maintain physicochemical stability and properties such as antimicrobial, antitumor, antioxidant, and antiproliferative activity. Also, the nanoencapsulation process can promote a better intestinal release rate, improve bioaccessibility and bioavailability, inhibit degradation in the gastrointestinal tract, ensure storage stability and color protection, apply in food packaging, promote thermal stability, and enable the oral administration of these compounds.

Some methods are indicated as viable for elaborating polysaccharide-based nanostructures to encapsulate polyphenols. The definitions, advantages, and applicability of the main techniques used to encapsulate polyphenols will be discussed below. (1) Emulsification/Nanoemulsion: this nanoencapsulation process involves mixing two immiscible liquids using an interface agent, such as a surfactant. Among the main advantages is the promotion of adequate solubility and kinetic stability, providing a sufficient matrix for targeted delivery, protection, and stability of polyphenolic compounds, which tend to be spherical droplets. However, using surfactants and co-surfactant can be potentially toxic, which may be one of the disadvantages of this technique (235–237). (2) Ionic gelation: biopolymers with electric charges can interact by different forces when homogenized in an aqueous solution forming nanostructures with different characteristics of isolated initial compounds. The polysaccharide can be dissolved in aqueous acid and added to a polycationic solution. The ionic-gelling polysaccharides can be precipitated to form spherical nanoparticles. The methodology can form stable nanostructures, and the low cost and non-addition of potentially toxic products are advantages of this technique. On the other hand, the disadvantages are the non-uniformity of size, and the release of the encapsulated compound may be limited (200, 238, 239). (3) Ionic cross-linking: formed by complexes of polyelectrolytes and complex coacervates. Nanoparticles are formed through the binding of divalent cations, such as the addition of calcium. Ionic cross-linking is based on the interaction between tripolyphosphate anions and protonated amine groups of polymers. The interaction occurs between two oppositely charged molecules or polyelectrolytes. Polysaccharide, mainly composed of guluronic acid and the mannuronic acid units (alginates), forms ionic complexes with divalent cations like Mg^2+^, Ca^2+^, and Ba^2+^. This technique forms stable molecules without adding toxic products and with reduced diameter sizes (200, 240, 241). (4) Coacervation: This process involves an interaction between two oppositely charged biopolymers forming a complex to protect a bioactive compound. This technique’s use of biodegradable compounds, adequate stability, and high encapsulation efficiency is advantageous. However, the complexity of elaboration and control of all the factors involved limits its application (238, 242, 243). (5) Self-assembly: different biopolymers are homogenized in an aqueous solution interacting by different electrostatic forces. It is low-cost and free of potentially toxic products. Despite this, encapsulation efficiency can be limited (62, 215, 238, 244). (6) Nanoprecipitation: the biopolymer nanoprecipitation process occurs by adding a non-solvent to a polymeric solution in supersaturation, nucleation, growth by condensation, and coagulation. It forms nanoparticles or polymeric aggregates that can adequately encapsulate polyphenolics. Despite having a limited controlled release, this method has low cost and broad applicability (245, 246).

Current challenges, such as increased chronic disease incidence in the population and diverse environmental impacts, instigate the research community for emerging intervention possibilities. There is considerable food waste from industries considered a potent source of nutritional compounds. In addition to the environmental impact of solid accumulation, there are significant economic losses. In this sense, nanotechnology can be an advantageous alternative. The development of functional nano foods could increase the consumption of bioactive compounds – extracted from food waste – generating a protective effect on public health. The use of food waste directs it to a noble destination and generates economic value (247, 248).

## 6. Nanocarriers based on natural polysaccharides: the promising interaction between pectin and phenolic compounds

Polyphenolic compounds encapsulated into pectin nanocomplexes for physical–chemical stabilization are a breakthrough in allowing several technological applications aiming at human health benefits (249). The functional properties (mainly antioxidant activity), bioaccessibility, and bioavailability of polyphenolic compounds are known to be influenced by compounds linked to their structure (250). Nanoencapsulation is not different, and physical–chemical studies should be done to depict how phenolic compounds are disposed inside the nanostructures to indicate determined applications since polyphenol-carbohydrate interactions can preserve chemical properties and stabilize the molecular structure (251). Moreover, pectin-polyphenolic interactions are influenced by these two compounds’ high structural variability and complexity (252, 253). Considerable scientific evidence indicates that polyphenol-carbohydrate interaction, especially with pectin, can preserve the properties of the polyphenols maintaining their chemical structure, inhibiting the degradation, and preserving the antioxidant action (101). Polyphenolic compounds and complex polysaccharides have a natural affinity and can bind via non-covalent and covalent interactions (107). Due to its characteristics, pectin has been used as a carrier to protect and deliver unstable bioactive compounds (209, 219).

Pectins are a class of polysaccharides composed of long chains of galacturonic acids widely distributed in nature (187, 254). Pectin is formed by heterogeneous non-starch polysaccharide complexes, originating from the structure of the cell walls of plant tissues and being considered an essential dietary fiber in the human diet (255). Pectins are formed by homogalacturonans (HG), type I (RG-I), and type II (RG-II) rhamnogalacturonans, in addition to xylogalacturonans and apiogalacturonans. Homogalacturonans are formed by linear structures of [→ α-1,4-D-galacturonic acid →], with variation in the degree of acetyl- and methyl esterification and may contain xylose (xylogalacturonan) (207, 256). Due to the possibility of ionization of galacturonic acids, pectins have good solubility in water. When galacturonic acid residues are esterified by methyl or acetyl groups, the solubility characteristics are modified according to the degree of methylation (high degree of methoxylation >50%, low degree <50%) (257). These compounds can produce highly viscous gels (depending on the degree of methoxylation and ligands), so they are used as an emulsifier and/or thickener (258). At neutral pH, the carboxylic acid (pKa pH ~3.6) has a net negative charge interacting with cationic molecules (proteins and polysaccharides) to form nanocomplexes (8). Pectins have the property of interacting with certain types of proteins at variable pH, stabilizing the nanostructures formed in acid or neutral dispersions (8, 213).

Due to their resistance to stomachal pH and human enzymes, pectins can protect and control the release of bioactive compounds, as they can reach (in their intact form) distal portions of the intestine, maintaining the functionality of different bioactive compounds nanoencapsulated in their structure (58, 136). In the intestine, pectins are fermented by microorganisms from intestinal microbiota. This fermentation process produces short-chain fatty acids with beneficial systemic effects and provides better absorption of nutrients and bioactive compounds (259). Due to its characteristics, pectin is a biomaterial suitable for forming nanocapsules for physical–chemical protection, increased absorption, and bioavailability of different active compounds. Using polyphenolic compounds as nanocarriers is an innovative alternative for designing new smart foods (23, 219).

Although the mechanism is not fully understood, it is known that polysaccharides, especially dietary fibers, can transport phenolic compounds in the gastrointestinal tract and protect them from the intestinal microbiota. The presence of these compounds in the intestinal lumen causes changes in intestinal bacteria composition through pectin fermentation (108, 260, 261). A recent study by Zhang et al. (261) aimed to evaluate the release and activity of polyphenols bound to soluble dietary fiber (wheat bran) in a simulated *in vitro* digestion and colonic fermentation system. The authors concluded that there was an influence on the bioaccessibility of fiber-bound polyphenols after colonic fermentation. There was a stimulation of the growth of beneficial bacteria after fermentation, indicating the potential prebiotic effect of the system.

The natural affinity of anthocyanins and polysaccharides was confirmed in a study that analyzed the tendency to bind during the processing of blueberry pomace with different dietary fibers, including pectin. A strong trend for anthocyanin-polysaccharide binding was observed (262, 263). The interaction occurs through electrostatic interaction between hydroxyl groups (OH^+^) and the carboxylic acids (COOH^−^) of the polysaccharides, including pectins (264). The different binding between anthocyanin and pectin was investigated in a study by Fernandes et al. (252). The interaction between cyanidin-3-*O*-glycoside and four citrus pectic fractions was explored through analyzes such as isothermal titration calorimetry, nuclear magnetic resonance, and UV–Visible spectrophotometry. The results indicated that different binding affinities could be correlated with changes in coloration, with the degree of pectin esterification being the primary determinant for complex formation.

Pectin-based nanostructures can be formed through different methods, such as spray drying, emulsion, hydrogel formation, liposomes, and nanocomplexes through electrostatic complexation and molecular self-organization. Pectin at neutral/basic pH predominates negative electrical charge and may interact with other positively charged macromolecules forming stable nanostructures (265, 266). Currently, pectin combined with other compounds (proteins, lipids, and polysaccharides) is considered a highly effective wall material for phenolic compounds (205). Stabilized polyphenolic compounds maintain color, functional properties, and stability in the gastrointestinal system improving their bioavailability (267, 268). Pectin is also considered a nanocarrier of polyphenolic compounds for the controlled release in distal parts of the colon, protecting from microbial processing from intestinal microbiota and promoting the absorption of intact molecules (200, 269).

## 7. Conclusion and future perspectives

Nanotechnology is a viable option to enhance the use of bioactive molecules from commonly wasted sources, producing a new product with high added value and broad applicability. Polyphenolic compounds benefit human health and protect against chronic non-communicable diseases, such as cardiovascular diseases, obesity, diabetes, and cancers. Using sustainable sources to extract phytochemicals is an effective strategy to reduce the accumulation of waste from the food industries on the environment. Fruit residues are sources of phenolic compounds and pectin. Pectin is an excellent encapsulating biomaterial for phenolic compounds aiming for innovative applications. Pectin-based nanostructures can protect compounds from molecular degradation and enable the development of nano-engineered foods for different purposes and applications. Applied nanotechnology adds economic value to these functional ingredients and reduces the impacts caused by the food industries.

**Figure 3:**
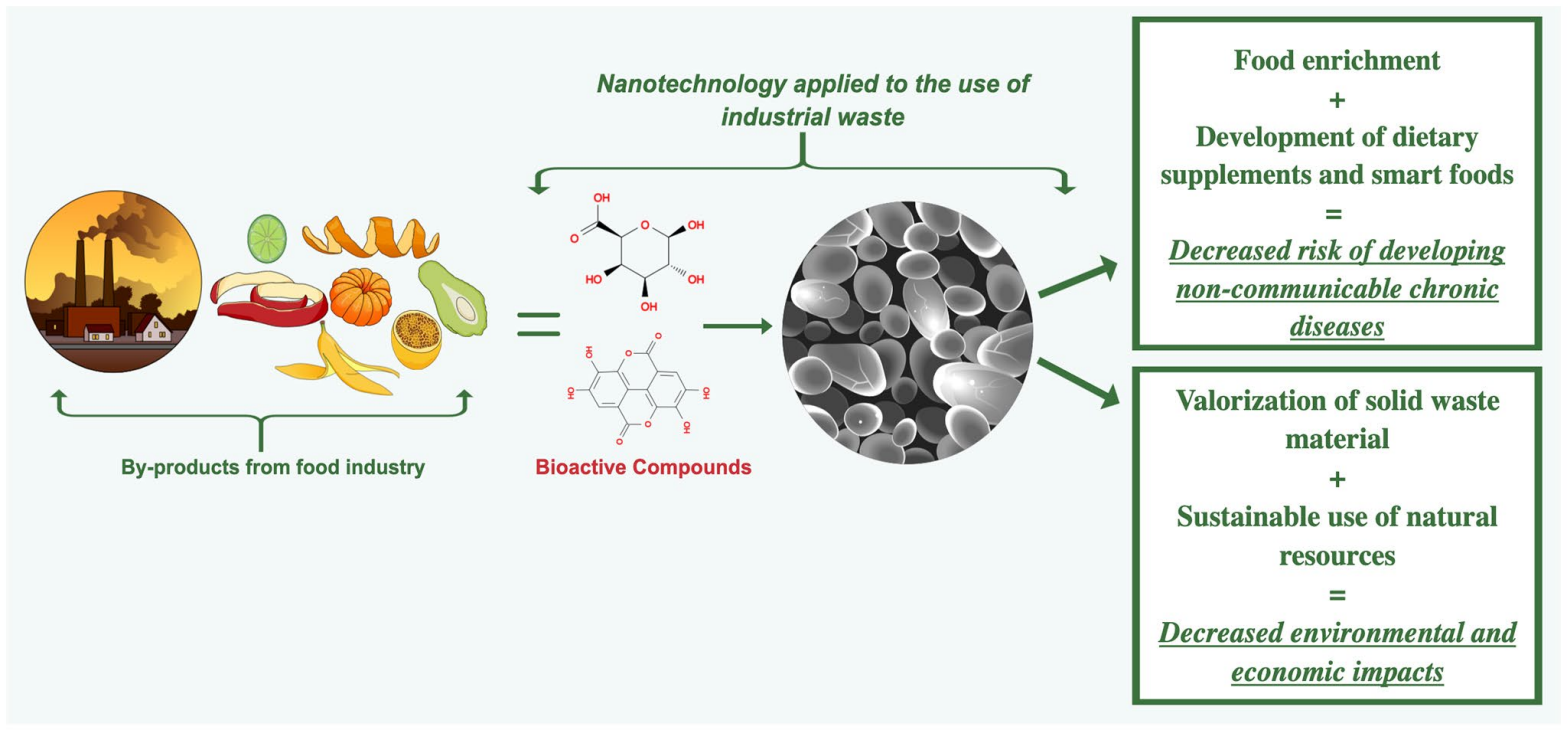
The by-products of the food industry can be used to extract bioactive compounds to be nanoencapsulated with polysaccharides also extracted from by-products. Food enrichment and new dietary supplements can be done to decrease the development of non-communicable chronic diseases, thus increasing the value of waste material and reducing environmental and economic impacts. The figure was created by Mind the Graph (https://mindthegraph.com) (accessed on 10 April 2023).

As discussed in the manuscript, it is essential to understand that despite being potentially applicable to nanotechnology in the valorization of industrial waste – positively affecting the circular economy and as a sustainability strategy – studies are strongly supported and necessary to attest to *in vivo* safety for human consumption. Another relevant factor is the ingested doses. Most of the results were performed in the laboratory using *in vitro* and *in vivo* models. Nanocapsules with phenolic compounds may enhance their bioavailability, so they should be carefully evaluated in toxicity studies (*in vitro* and *in vivo*) and long-term ingestion. Also, another fundamental point that must be considered is the optimization for production on an industrial scale; from the extraction process to the elaboration of nanostructures, these parameters still need to be explored. This review provided new perspectives for possible research directions in nanoscience and minimizing impacts on nature, as well as supporting information for using different fruit peels rich in polyphenolic compounds to be extracted with potential benefits for human health. Many efforts have been made to mitigate food waste and protect the environment. However, innovative ideas with practical strategies for fully utilizing industrial by-products are urgently needed. Using nanoencapsulation of polyphenolic compounds extracted from by-products characterizes the circularity in food systems, promoting new functionality for products with no commercial value while encouraging the increased consumption of bioactive compounds. Dietary supplements and foods enriched with nanoencapsulated bioactive compounds are considered promising to reduce the risk of developing various diseases. The use of polyphenolic compounds within nanocapsules represents new perspectives on current study gaps and future directions in this field, providing enriched and highly specialized foods for optimal target intestinal release of bioactive compounds. The full use of by-products is achieved through technological incentive policies that support the use of unused parts of plants to be recovered and destined for the full use of their nutritional, techno-functional, and health-enhancing properties, resulting in economic, environmental, and public health benefits. The impact on the valuation of losses/waste and by-products of the food industry through the application of nanotechnology represents opportunities, trends, and innovations (Figure 3).

## Author contributions

TR: conceptualization, data curation, and writing – original draft and review and editing. JF: conceptualization, supervision, writing – review and editing, and grant acquisition. All authors have read and agreed to the published version of the manuscript.

## Funding

The authors acknowledge The National Council for Scientific and Technological Development (CNPq) for JF productivity scholarship (CNPq Proc. #307842/2022-3). The study was financially supported by grants #2013/07914-8 and #2019/11816-8 from the São Paulo Research Foundation (FAPESP).

## Conflict of interest

The authors declare that the research was conducted in the absence of any commercial or financial relationships that could be construed as a potential conflict of interest.

## Publisher’s note

All claims expressed in this article are solely those of the authors and do not necessarily represent those of their affiliated organizations, or those of the publisher, the editors and the reviewers. Any product that may be evaluated in this article, or claim that may be made by its manufacturer, is not guaranteed or endorsed by the publisher.
